# Retro-version and Retro-flexion of the Uterus

**Published:** 1854-07

**Authors:** 


*Retro-version and retro-flexion of the Uterus.*

For these, Dr. Simpson relies on his stem-pessary. In a case just arrived from Aberdeen, the patient was placed deeply under the influence of chloroform, the misplacement clearly ascertained, and as the os tincæ would not admit the stem, it was freely incised in opposite directions. The patient was to return in a few days to have a pessary adapted to her case. I was much surprised at these bold operations upon the womb, and they go far to establish the position of Jobert, of Paris, that the os tincæ is insensible. The instrument used by Prof. S. for stricture of these parts, resembles the lithotome caché, the handle being much longer.

*False Conception* is readily detected by the relaxation produced in the abdominal muscles from the effect of chloroform.—*American Medical Monthly.*

